# Rasch analysis of the patient-rated wrist evaluation questionnaire

**DOI:** 10.1186/s40945-018-0046-z

**Published:** 2018-02-26

**Authors:** Saravanan Esakki, Joy C. MacDermid, Joshua I. Vincent, Tara L. Packham, David Walton, Ruby Grewal

**Affiliations:** 1 School of Physical Therapy, Western University, London, ON Canada; 2 School of Rehabilitation Science, McMaster University, Hamilton, ON Canada; 3 The Hand and Upper Limb Centre, St Joseph’s Health Centre, London, ON Canada; 4 Department of Surgery, University of Western Ontario, London, ON Canada

**Keywords:** PRWE, Rasch analysis, Patient-reported outcome measure, Distal radius fracture

## Abstract

**Background:**

The Patient-Rated Wrist Evaluation (PRWE) was developed as a wrist joint specific measure of pain and disability and evidence of sound validity has been accumulated through classical psychometric methods. Rasch analysis (RA) has been endorsed as a newer method for analyzing the clinical measurement properties of self-report outcome measures. The purpose of this study was to evaluate the PRWE using Rasch modeling.

**Methods:**

We employed the Rasch model to assess overall fit, response scaling, individual item fit, differential item functioning (DIF), local dependency, unidimensionality and person separation index (PSI). A convenience sample of 382 patients with distal radius fracture was recruited from the hand and upper limb clinic at large academic healthcare organization, London, Ontario, Canada, 6-month post-injury scores of the PRWE was used. RA was conducted on the 3 subscales (pain, specific activities, and usual activities) of the PRWE separately.

**Results:**

The pain subscale adequately fit the Rasch model when item 4 “Pain - When it is at its worst” was deleted to eliminate non-uniform DIF by age group, and item 5 “How often do you have pain” was rescored by collapsing into 8 intervals to eliminate disordered thresholds. Uniform DIF for “Use my affected hand to push up from the chair” (by work status) and “Use bathroom tissue with my affected hand” (by injured hand) was addressed by splitting the items for analysis. After background rescoring of 2 items in pain subscale, 2 items in specific activities and 3 items in usual activities, all three subscales of the PRWE were well targeted and had high reliability (PSI = 0.86). These changes provided a unidimensional, interval-level scaled measure.

**Conclusion:**

Like a previous analysis of the Patient-Rated Wrist and Hand Evaluation, this study found the PRWE could be fit to the Rasch model with rescoring of multiple items. However, the modifications required to achieve fit were not the same across studies, our fit statistics also suggested one of the pain items should be deleted. This study adds to the pool of evidence supporting the PRWE, but cannot confidently provide a Rasch-based scoring algorithm.

**Electronic supplementary material:**

The online version of this article (10.1186/s40945-018-0046-z) contains supplementary material, which is available to authorized users.

## Background

Patient-reported outcome measures have become a cornerstone of evaluation in hand therapy and hand surgery [1, 2]. A well-developed patient-reported outcome measure (PROM) can provide a clinically relevant evaluation of the patient perspective and status to inform health care decisions [1, 2]. Classical test theory (CTT) forms the basis for most evaluations of measurement properties [3]. A core tenet of CTT is validity and reliability results apply only to the sample studied [4]. This has resulted in a proliferation of studies on commonly used outcome measures in upper extremity rehabilitation to evaluate measurement properties in a spectrum of contexts and patient populations [5].

Rasch analysis (RA) is a form of mathematical modeling employed to develop new outcome measures and appraise the properties of existing instruments [6]. RA extends the measurement evaluation by critically evaluating discrete items and scores, which is an advantage over CTT [6]. The Rasch rating scale is based on item response theory (IRT) [7]. However, according to its developer George Rasch, the central differentiating feature between the Rasch and IRT is the defining role of specific objectivity, which presumes both individuals and items can be rated [8]. Rasch uses probabilistic modeling to determine the degree to which items on a scale function as linear (interval-level) measurement of the latent construct, or domains of interest. Further, it models the predicted amounts of this latent construct within the individuals studied [9, 10]. While this interval-level of measurement is a pre-requisite for much statistical analysis, many scales developed within CTT fail to meet this interval measurement standard and are used for decision making and statistical purposes, this may ultimately influence the validity of research findings [5].

The Patient-Rated Wrist Evaluation [PRWE] [11] (see Additional file 1) is a patient-reported outcome measure intended to quantify perceptions of pain and disability evolving from wrist conditions. The PRWE has been used in more than 150 studies and has been recommended as a core measure for evaluating outcomes in distal radius fracture (DRF) [12]. While the PRWE questionnaire was originally developed for distal radius fracture, the scoring instructions (but not the items) were later modified as the Patient-Rated Wrist and Hand Evaluation [PRWHE] [12, 13] to address not only wrist but also hand conditions. A recent systematic review summarized 22 studies examined measurement properties of the PRWE and found strong supporting evidence for reliability and responsiveness [14]. The validity of the PRWHE version is supported for the use in patients with wrist and hand conditions by demonstrating similar responsiveness with the components of the Disabilities of the Arm, Shoulder, and Hand (DASH) [15], but the systematic review noted a gap in clinically relevant indicators like the minimally significant difference. While triangulating findings from different measurement models such as CTT and Rasch can lend confidence to the properties of a scale, only a single study reported the properties of the instrument using RA. Packham and MacDermid conducted RA on the PRWHE using 264 patient records representing a mixture of wrist and hand injuries and found good to excellent reliability of the scale [16]. The study found no significant differential item functioning, or scale differences between the people with injuries in the dominant hand when compared to the injuries in the non-dominant hand. A key finding was best fit to the Rasch model was attained if the disability scale was split into the specific activities and usual activities components for analysis [16]. Background rescoring of some items was also required: however, the authors cautioned that revising the PRWHE based on a single study would be premature. A critical limitation of this study was some patients were represented multiple times (at different time points in their recovery) within the dataset. Another concern limiting the application of the findings in the study did not address the work status of the participants [16]. Return to work status has been used for many years by rehabilitation professionals as an objective indicator of function, and is an increasingly popular measure of overall recovery from injury [17, 18].

Given the return to work is considered a vital indicator of recovery [19], current work status (compared to pre-injury) may be an important person factor upon which to evaluate the measurement properties of a questionnaire. While there is no reason to believe the difference in wording of the instructions (the only difference between the PRWHE and PRWE is ‘wrist/hand’ is substituted for ‘wrist’ on the PRWHE) would substantially influence measurement properties, there is an opportunity to replicate the Rasch analyses of the PRWHE using the PRWE. Furthermore, RA of PRWE data gathered in a different practice setting and from a different patient population would provide an opportunity to contrast and compare the stability of findings in the different practice environment. Although RA is thought to be less affected by participant samples, this has not always been empirically supported [20, 21]. Taking these considerations together, there is a need to further explore the measurement properties of the PRWE using the Rasch paradigm with a different population.

The purpose of this study is to utilize RA to evaluate the PRWE in a cohort of persons following distal radius fracture:

1) To test the construct validity of the pain and disability subscales of the PRWE by examining the unidimensionality of the scales, and to evaluate the reliability as defined by Rasch traditions.

2) To examine the interval-level properties of the pain and disability scales of the PRWE by examining the fit to the Rasch model and ordering of item thresholds.

3) To examine the potential for bias in PRWE score based on age, gender and work status of respondents, and to explore solutions for minimizing any bias.

## Methods

### Research design

#### Cross-sectional study using RA

##### Instrument and procedures

The 15-item PRWE questionnaire evaluates the domains of pain and disability with three subscales: pain, specific activities, and usual activities. The pain subscale has five items, rated as 0 = no pain to 10 = worst pain ever. Functional interference, or disability, is represented by six items on a specific activities subscale and four items on usual activities: these are rated on a 0–10 scale where the subject scores the amount of difficulty in performing the activity, with 0 = no difficulty in performing an activity and 10 ‘unable to perform the activity’. The final PRWE score represents equally weighted pain and disability (function) scores. We selected the 6-month post-injury scores for analysis as a return to work status was also evaluated at this time point.

## Data collection

### Sample size and characteristics

We conducted a secondary analysis of a convenience (existing) cross-sectional data set consisting of 300 and 82 patient’s PRWE scores 6 months post-injury, collected at the Hand and Upper Limb clinic at a large academic healthcare organization in London, Ontario, Canada. Ethics approval for the original study was received from Western University ethics board. Men and women accounted for 32.5% and 67.5% of the sample respectively: mean age was 58.5 years. 67% (*n* = 256) were working and 33% (*n* = 126) were non-working population. 19.6% (*n* = 78) participants have left hand as dominance and 61.2% (*n* = 234) participants have DRF at the dominant side hand (right or left). RA requires large samples to ensure adequate distribution of responses for analysis across all levels of the condition or construct of interest: a minimum of 250 or at least 10 endorsements for each potential response category for each item have been suggested as standards for ensuring adequacy of sample size [21].

The paper copy of the PRWE questionnaire was completed by the patients, and the data were compiled in SPSS for demographic examination and then imported into the RUMM2030 version 5.1(RUMM Laboratory Pty Ltd., Perth, Australia) [22] for RA.

### RA

We followed the approach suggested by Lundgren Nilsson and Tennant [6] as described below. The 3 subscales of the PRWE were analyzed separately for sources of misfit to the model [23, 24]. Bonferroni correction [25] was applied throughout the analysis to reduce alpha errors due to multiple testing.

### Likelihood ratio (LR) test

The choice of Rasch model (Rating Scale vs. Partial Credit) [24] was made by conducting and interpreting a likelihood ratio test that evaluates the likelihood that mathematical differences between polytomous response options are equal. A significant LR suggests they are not equal, and that the unconstrainted partial credit model should be used [24].

### Class interval and distribution structure

The size of class intervals was checked throughout the analyses to ensure equal distribution between the intervals. Class intervals are generated by the analysis software after ranking the person location: the sample is then split into relatively equal class intervals to ensure adequate representation of the key patient variables in each class for differential item functioning analysis [26]. In this instance, 4 class intervals were generated.

### Thresholds

Category probability curves were used to identify disordered thresholds, item misfit and inconsistent use of items. Disordered thresholds arise when the respondents find it difficult to differentiate between the item response options [27]. This occurs when there are too many response options, or the selection options are similar to one another, confusing or open to misinterpretation. Disordered thresholds can be corrected either by rescaling the tool or by collapsing the categories and revising the response option to improve the overall fit to the model [27].

### Fit statistics

The following important fit statistics are inspected when analyzing the fit of the data to the Rasch model.

### Unidimensionality

Unidimensionality is one of the main assumptions for the data to fit the Rasch model. The absence of any meaningful pattern in the residuals reveals the presence of unidimensionality [6]. A test proposed by Smith [28] examines the relationship between items and the first residual factor identified by principal components analysis and uses these patterns to define 2 subsets of items. By then testing using paired t-tests, we could see if the person estimate derived from these subsets significantly differs from that derived from all items. For the questionnaire to be unidimensional, the percentage of tests that are significant (*P* < 0.05) should be less than 5%. Final evaluation of unidimensionality is completed only when all other scale adjustments have been completed [28].

### Local dependency

RA employs Principal Components Analysis (PCA) of the residuals to ensure the local independence of the items [6]. An inter-item residual correlation > 0.2 above the average residual correlation was used as the threshold to indicate local dependency [6].

The local independence assumption can be violated in 2 ways: response dependency, and multidimensionality [29]. Response dependency occurs when the items are linked in such a way that the response on one item determines the response on another. These dependencies can be identified by the residual correlation matrix and rectified by combining the items into a ‘super item’ by combining 2 items or by deleting one of the dependent items. Again, subsequent reanalysis is required to confirm resolution of the dependency [8].

### Item/person fit residuals

In RA, the scale is always centered on zero logits, which represents the average item difficulty for the scale. Individual item fit is then calculated relative to this point, or are ‘fitted’ to the model. Person fit is then evaluated by considering the mean location of persons in the sample. For a well-targeted measure, the mean location for persons would be around the value of zero. When the mean is approximately zero, and the standard deviation is close to one, the item and persons (residuals) fit the model, and a hierarchical ordering of items (e.g., from low to high levels of activity limitation) is achieved [4, 30]. Standardized fit residuals for individual items were flagged as extreme if the values exceeded + 2.5.

### Item-trait interaction

To analyze the property of invariance across the trait being measured, item-trait interaction is assessed using a chi-square statistic. The chi-square statistic [31] compares the difference in observed values with expected values across groups representing different ability levels (called class intervals) across the trait to be measured (e.g., pain). A significant chi-square value (< 0.05) indicates that the hierarchical ordering of the items varies across the trait, compromising the required property of invariance [31].

### Reliability indices

Person-Separation-Index (PSI) indicates the reliability of the scale for estimating the amount of latent trait in any individual. This can also be interpreted as the ability of the scale to identify differences among respondents [32]. A person-separation value of a minimum of 0.7 and maximum of 0.95 is considered, in general, to be the acceptable level of PSI. Reliability of the fit characteristics depends on the value of the PSI, with higher PSI indicating higher reliability [32].

### Differential item functioning (DIF)

DIF indicates potential sources of bias in-person measurements which result in misfit of the data to the Rasch model. DIF occurs when distinct subgroups within the sample population respond in divergent ways to the individual item even though they have equal levels of underlying characteristics [8]. DIF can be identified both graphically, by analyzing the item characteristic curves, and statistically using analysis of variance (ANOVA) [33]. DIF can occur in two forms: uniform and non-uniform. Uniform DIF occurs when the difference in scoring performance remains constant across all respondent’s ability levels. Uniform DIF can be rectified by either combining items or by splitting items: subtest analyses are performed to verify the DIF was canceled by these adjustments. Non-uniform DIF occurs when the difference in performance varies with the level of the attributes. There is no definite procedure to rectify the non-uniform DIF, and therefore, the most common solution is for that item to be re-written or removed from the questionnaire [6]. In this RA, we tested DIF on working status, injured hand, dominant hand, gender and age group variables.

Item difficulty Item difficulty for each of the subscales was graphically represented by the generation of a Wright map [10]. This allows the difficulty of the items to be compared and serves as a form of content validation by looking for potential floor or ceiling effects.

## Results

In this study, the likelihood ratio was statistically significant (*P* value < 0.05): therefore, we used partial credit parameters for the analysis [24]. There were no missing data, and all 382 independent cases were determined to be valid by the RUMM 2030 software. The 3 subscales were analyzed separately, as we presumed each subscale represented a distinct latent trait.

### Pain subscale

Initial analysis of the 5 items on the pain subscale using the partial credit model showed shows excellent Individual item fit and indicated acceptable levels of discrimination. Item 5 (How often do you have pain?) shows disordered threshold. The scale also shows significant item-trait interaction (*p* < 0.001) and PSI of 0.84. Item 4, “Pain at its worst” showed non-uniform DIF across age groups. Local dependency was observed between item 1 and 5, where predictably, persons with pain at rest reported high frequencies of pain. Acceptable unidimensionality was observed. (Table 1: initial analysis).

**Table 1:** Summary fit statistics for individual subscales of the PRWE^a^

Analysis	Item fits residual		Person Fit residual		Item-trait interaction		Unidimensionality	PSI^a^
	Mean	SD	Mean	SD^a^	Chi-square *(df)*^*a*^	*P*	Per C < 5%^a^	
PAIN SUBSCALE								
Initial	−0.41	1.72	−0.45	0.94	23 (20)	0.49	6.9%	0.89
Final	−0.53	1.17	−0.44	0.91	18 (20)	0.54	2.5%	0.84
SPECIFIC ACTIVITIES SUBSCALE								
Initial	−0.60	2.13	−0.45	1.07	54 (30)	0.00	4.7%	0.81
Final	−0.47	1.87	−0.37	1.03	32 (25)	0.04	- (since items were split for DIF)	0.80
USUAL ACTIVITIES SUBSCALE								
Initial	−0.41	1.72	−0.45	0.94	23 (20)	0.28	1.5%	0.78
Final	−0.43	1.58	−0.45	1.00	35 (25)	0.07	- (since items were split for DIF)	0.81

^a^Source of misfit to the Rasch model; SD = Standard deviation; df = Degrees of freedom; per C < 5% = proportion of t-tests that were significant at level of significance of 0.05; 95% CI = 95% confidence interval; PSI = Person separation index; PRWE – Patient-Rated Wrist Evaluation

For the data to satisfy Rasch model requirements:

The mean is expected to be approx. Around zero (Can range between ± 2.5);

S.D. should be approx. 1;

Chi-square value is expected to be small and statistically non-significant;

For a measure to be unidimensional per C < 5% should be less than 0.05; if it is higher than 0.05 then look into the lower limit the 95% confidence interval if it is less than 0.05 then the measure is unidimensional

**Fig. 1:**
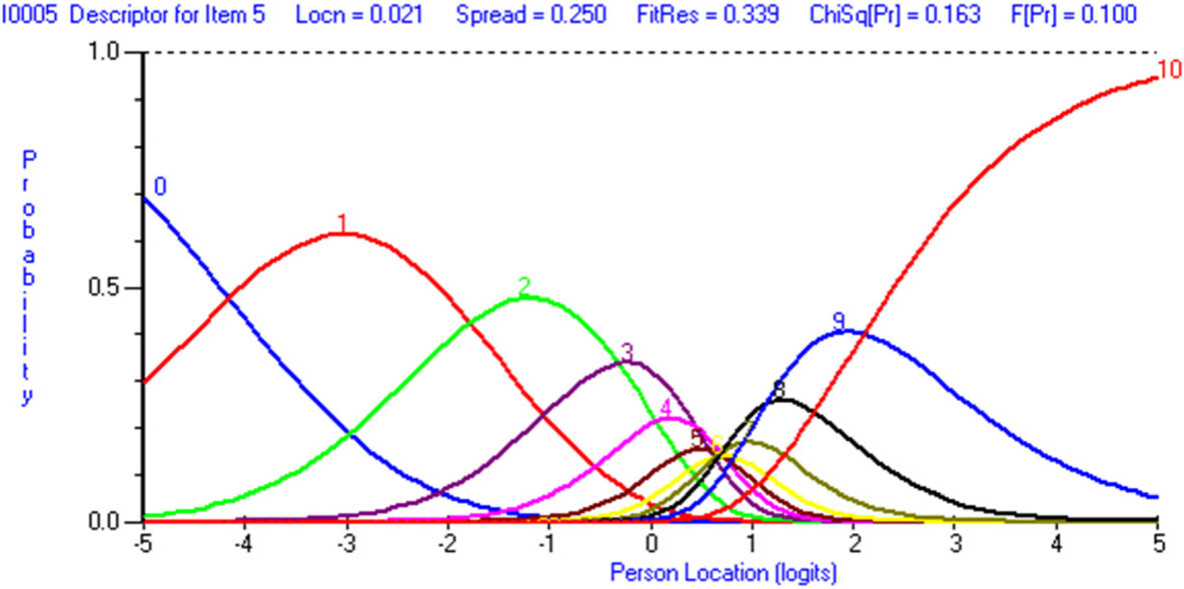
Pain item 5 (Pain frequency) category probability curve

**Fig. 2:**
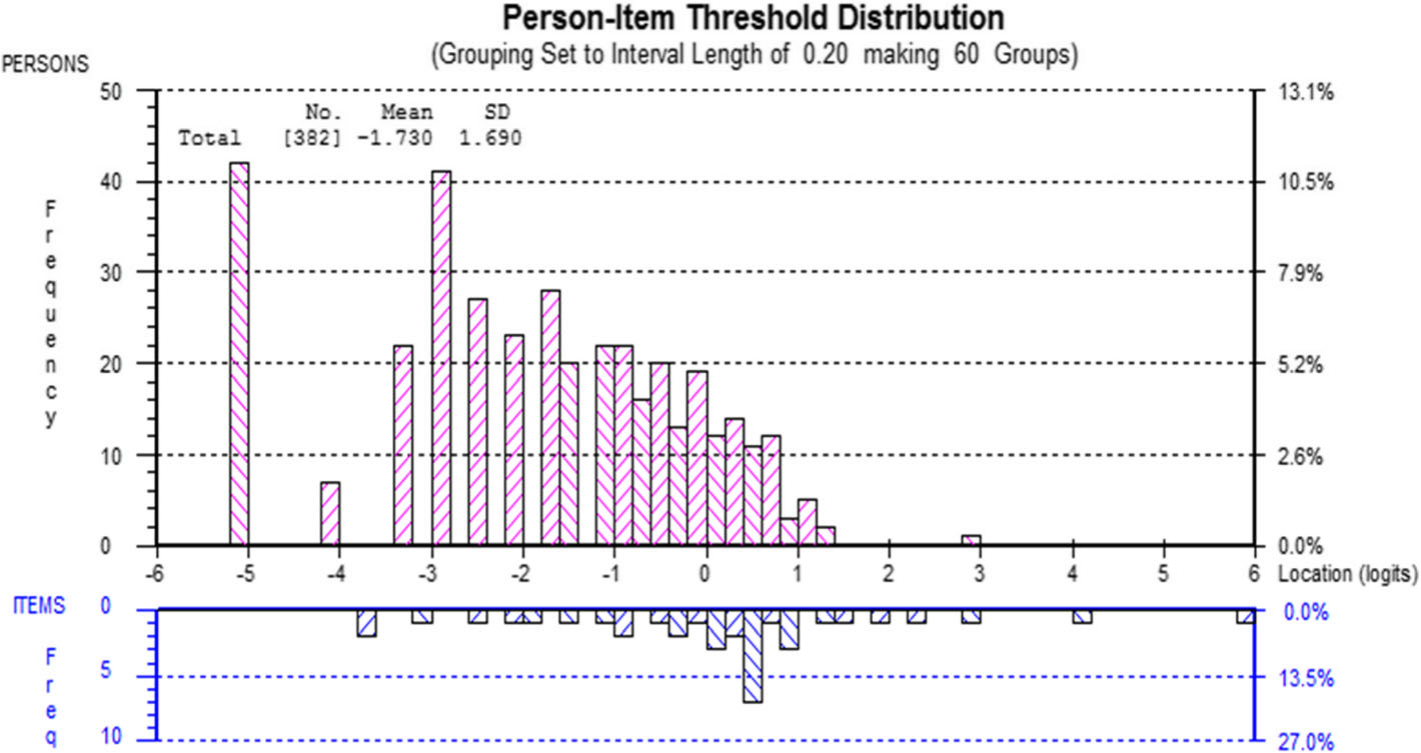
Person Item threshold (Pain subscale)

PSI (Person separation index) should be greater than 0.70 for the summary statistics to be reliable;

To improve the overall fit to the Rasch model item 5 was rescored by collapsing response categories from the original 0–10 responses based on the category probability curves (Fig. 1) until the rescored curves show no disordered thresholds (F = 2.056, df 3, *p* = 0.105931), resulting in 8 categories (Table 2). Item 4 was deleted to eliminate the Non-Uniform DIF (Age group) (F = 2.290, df 3, *p* = 0.078249). Re-analysis after rescoring shows that the person-item threshold map (Fig. 2) indicates that this subscale has good item coverage for wrist disorders related pain. Also, no local dependency was present and unidimensionality was observed.

**Table 2:** Table showing the structure of scores for individual items of the PRWE

Item	0	1	2	3	4	5	6	7	8	9	10
PAIN SUB SCALE											
Pain - At rest	0	1	2	3	4	5	6	7	8	9	10
Pain - When doing a task with repeated wrist/hand movement	0	1	2	3	4	5	6	7	8	9	10
Pain - When lifting a heavy object	0	1	2	3	4	5	6	7	8	9	10
Pain - When it is at its worst	0	1	2	3	4	5	6	7	8	9	10
How often do you have pain?^a^	0	1	2	3	3	4	4	5	5	6	7
SPECIFIC ACTIVITIES SUB SCALE											
Turn a door knob using my affected hand	0	1	2	3	4	5	6	7	8	9	10
Cut meat using a knife in my affected hand	0	1	2	3	4	5	6	7	8	9	10
Fasten buttons on my shirt	0	1	2	3	4	5	6	7	8	9	10
Use my affected hand to push up from a chair	0	1	2	3	4	5	6	7	8	9	10
Carry a 10 lb. object in my affected hand	0	1	2	3	4	5	6	7	8	9	10
Use bathroom tissue with my affected hand	0	1	2	3	4	5	6	7	8	9	10
USUAL ACTIVITIES SUB SCALE											
Personal activities (dressing, washing)	0	1	2	3	4	5	6	7	8	9	10
Household work (cleaning, maintenance)	0	1	2	3	4	5	6	7	8	9	10
Work (your job or everyday work)^a^	0	1	2	3	3	4	4	4	5	5	6
Recreational activities^a^	0	1	2	3	3	4	4	4	5	5	6

^a^Rescored items; PRWE – Patient-Rated Wrist Evaluation

### Specific activities subscale

Initial analysis showed there are no disordered thresholds and the subscale demonstrated unidimensionality (Table 1: initial analysis). Good Individual item fit, a good level of discrimination and high reliability of the scale was observed (Table 1: initial analysis). The fit challenges included uniform DIF for item 4 (“Use my affected hand to push up from a chair”) (F = 15.769, df 1, *p* = 0.000091) and item 6 (“Use bathroom tissue with my affected hand”) (F = 0.183, df 1, *p* = 0.669405) by both work status and injured hand. None of the items exhibited DIF for gender or dominant hand. Local dependency was observed between the item 1 (“Turn a door knob using the affected hand”) and item 2 (“Cut meat using a knife in my affected hand”).

**Fig. 3:**
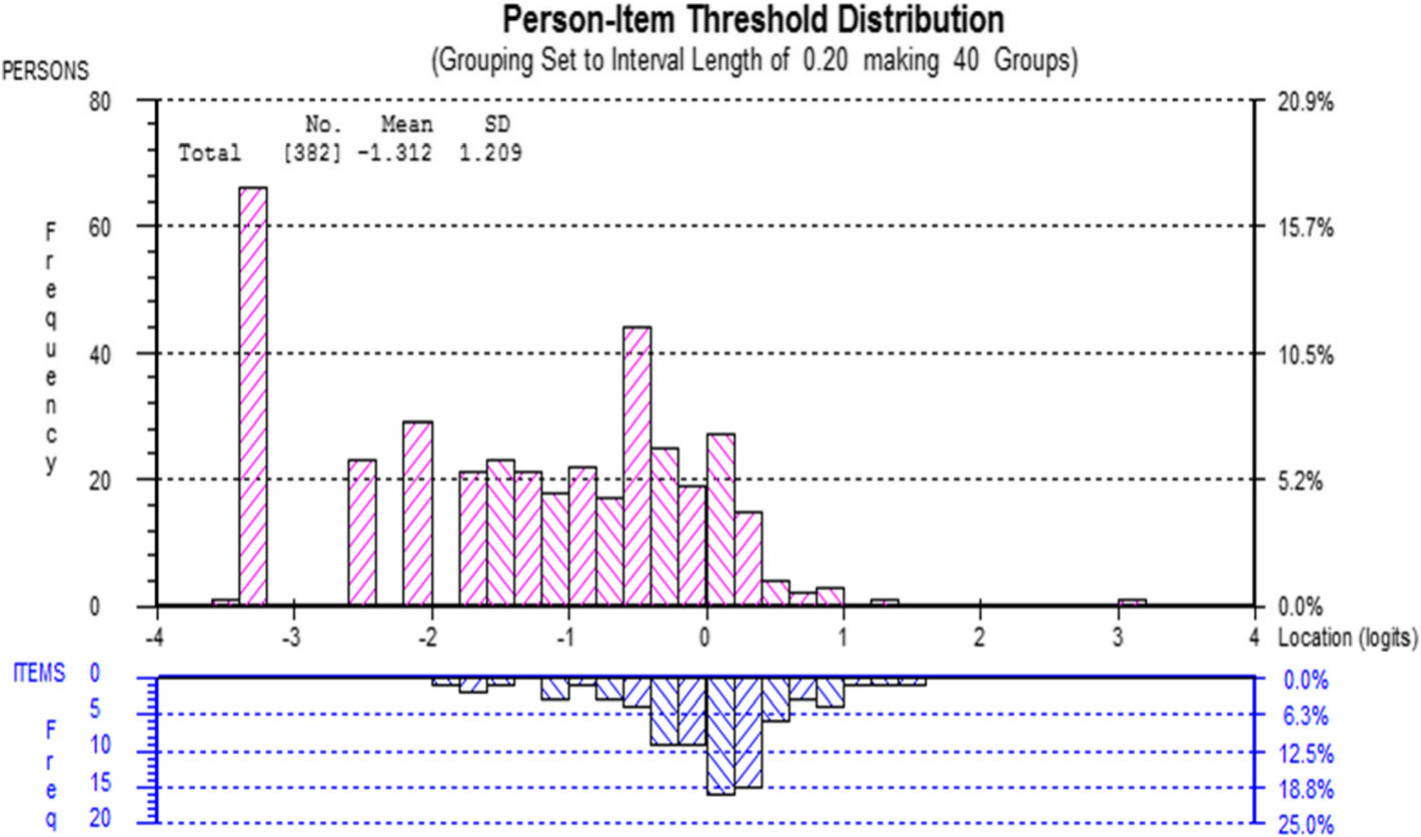
Person-item threshold distribution (Specific activities)

To improve the overall fit of the specific activities subscale to Rasch model, the following actions were taken. Initially, to deal with DIF, we split the item 4 for the work status as “Yes” and “No” for working and not working and split the item 6 for the injured hand as into right: left: both. This essentially creates a different PRWE scoring system for persons in each of these categories. Then item 1 and 2 were bundled (treated statistically as a single item) to address the local dependency. Re-analysis of the altered scale confirmed that DIF and local dependency were resolved. Chi-square residual became non-significant indicating an acceptable fit of the data to the Rasch model (Fig. 3), and the analysis showed unidimensionality (Table 1: final analysis).

### Usual activities subscale

The usual activities subscale analysis demonstrated that the scale was unidimensional with 95% confidence interval (0.0150) and reliability was good (PSI = 0.78) (Table 1: initial analysis). No DIF was observed for the injured hand, dominant hand, and gender. However, it shows misfit to the Rasch model with disordered thresholds for two of the four items (items 3 and 4). Item 2 “Household work (cleaning, maintenance)” showed uniform DIF for work status, while non-uniform DIF for age group was observed for item 4 “Recreational activities”.

**Fig. 4:**
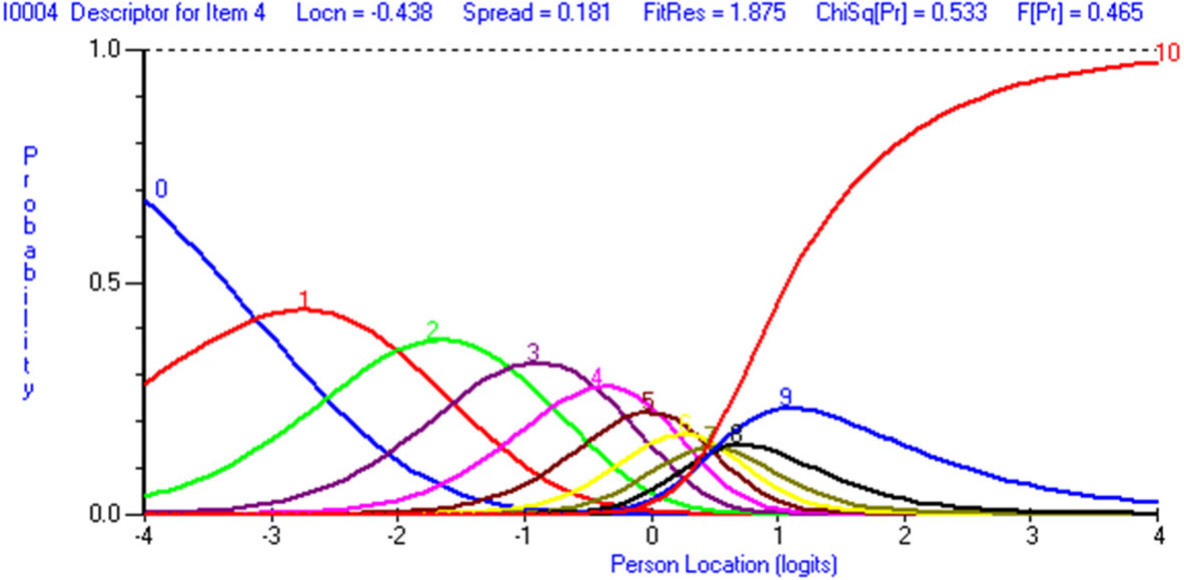
Usual activities category probability curve

**Fig. 5:**
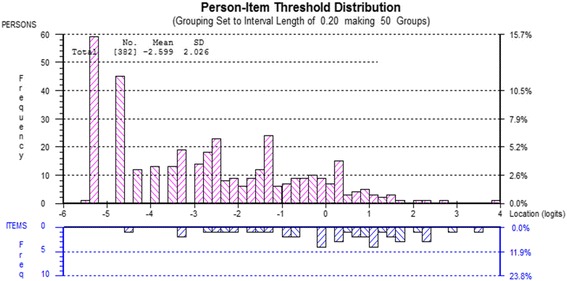
Usual activities Person-item threshold

To increase the overall fit of the scale to the Rasch model, the items (3 and 4) with disordered thresholds were collapsed to reorder into 7 intervals. (Table 2). After rescoring, summary statistics showed no local dependency on the scale. To eliminate the DIF, item 2 was split for work status as “Yes” and “No” for working and not working (F = 4.054, df 1, *p* = 0.045085), and item 4 was split for the age group as 0–35 years: 36–50 years: 51–65 years: 65 years plus for the better distribution of persons within this category (F = 1.693, df 3, *p* = 0.168899). The final analysis demonstrated the data to fit the Rasch model, increasing the reliability of the subscale (PSI = 0.86) and decreased chi-square value (Table 1: final analysis, Fig. 4). The person-item threshold map illustrated the high level of recovery seen in the sample of 6 months post recovery in DRF sample (pink bars). The scale has good coverage across the range of abilities, and that’s illustrated by the figures (blue bars) (Fig. 5).

## Discussion

The result of this RA adds additional evidence for acceptable measurement properties to that derived from classical test methods on the psychometric properties of the PRWE. As with many Rasch analyses conducted on instruments that were not developed using Rasch, some modifications were required to achieve interval level scaling or fit to the Rasch model. However, these adjustments indicated an adjusted PRWE could provide interval level scaling and appropriate targeting for a DRF population.

During the initial steps of our RA, we found three disordered item thresholds from the pain (1 item) and usual activity (2 items) subscales. Similar findings have been observed in the previous study on the measurement properties of the PRWHE (3 items) [16] and the analysis of the similarly structured Patient-Rated Elbow Evaluation (PREE) (17 items) [33]. Other work has suggested disordered thresholds in 0 to 10 scales may reflect difficulty for respondents in finding meaningful distinctions between 11 different response categories. Nonetheless, the concept of 0 to 10 is commonly used and easy for patients to understand. It is not clear if overtly rewriting items to have fewer categories would be beneficial, especially since the optimal number of categories would then vary by question. Therefore, background rescoring is a simple solution and is commonly used to reduce items responses to fewer categories “behind the scenes” without disturbing the original construction or outward appearance of the item that is in common use [34].

The three subscales of the PRWE were considered separately to accommodate the unidimensionality assumption of the Rasch model. Unidimensionality was observed in all the three subscales. This suggests each subscale represents a unique construct and would support comparisons between these components, as well as comparisons based on the total PRWE score. This is concordant with the previous PRWHE analysis [16], which was unable to fit the disability component to the model as a unit, and needed to look at the subscale level in order to see fit to the model. Confirmatory factor analysis of this dataset could potentially validate this perspective and is warranted. While it is common to use a single summary score in reporting outcomes in clinical studies, it has become more apparent that pooling different constructs can have drawbacks. Recent recommendations around the use of core measures suggest that pain and disability subscales should be considered as separate constructs [33]. Our RA approach is consistent with that recommendation. Given that trials will continue to prefer to use a single summary outcome measure, it is advisable that such studies also examine subscale differences in outcome to avoid inaccurate interpretation of the impact of interventions.

In the pain subscale, we found non-uniform DIF was exhibited based on age group. This misfit should be considered in the context that previous pain studies indicate the perception of pain differs from person to person and also pain tolerance is reduced as people age [35, 36]. A meaningful pattern of local dependency was observed between items 1 “pain at rest” and 5 (pain frequency). If the pain is reported at rest, the person is also likely to have pain more frequently. But these local dependency issues were accommodated when subtest analysis was performed, demonstrating the impact of this correlation is mitigated by always considering both items together in the context of the scale. In terms of clinical implications, it is important to note these adjustments we made to fit the PRWE to the Rasch model are to support the accuracy of interval level scoring [33] for research comparisons to other interval level variables such as grip strength.

In the specific activities subscale, uniform DIF was observed by work status for “use my affected hand to push up from a chair.” It may be that people in the workplace have more possibilities of using the injured hand while pushing up from the chair when compared to the non-working participants thus calibrating this differently. Similarly, in usual activities subscale, differential item functioning by age groups may reflect the difficulty in transferring that is often reported by older adults aged 65 and above [37]. However, work status and age are correlated in this sample which reflects the epidemiology of DRF, since most older adults aged 65 and above would not be working [38]. So, it is difficult to determine which factors drive the differential item functioning. Gender or dominant hand did not exhibit DIF, which shows that this scale has good construct validity. A dominance effect has been reported in some patient-reported outcome measures, such as the DASH [15], however, the PRWE instructions refer to “the affected hand”, whereas the DASH refers to difficulty at the person-level: this may account for the differences seen in the importance of dominance as an outcome mediator. The local dependency observed between the “Turn a door knob using the affected hand” and “Cut meat using a knife in my affected hand” has not been reported in other Rasch analyses of the PRWHE, PREE and is not intuitive. It may be that difficult with a hand grip that occurs following DRF links these items in this sample. Since the prior RA included different diagnoses, this link may not have occurred in a more heterogeneous sample. Because it has not been reported in previous studies, the dependency may not generalize beyond this sample.

In the usual activities subscale, “Household work (cleaning, maintenance)” was the source of misfit. The analysis shows uniform DIF for the work status: this may be due to the differences in the perception of the household work among the people who are working and who are not, and their contribution to household tasks. People who hold paid employment outside the home may contribute relatively less to household work when compared to people who stay at home [39]. This could explain the reason for observing a uniform DIF. This finding was similar to RA results reported for the PREE, where uniform DIF was observed on “Household work (cleaning, maintenance)” from the usual activities subscale for gender. In our sample, non-uniform DIF was observed between different age groups for “Recreational activities”, perhaps due to the age-based differences in participation as the type of activities, intensity of the recreational activities, and the value of participation in recreation also differs between the age groups [38, 40]. Younger adults often engage in high-intensity recreational activities while older adults may tend to do mild to moderate intensity recreational activities, but may perceive the level of intensity differently. Recreational activities in older adults are most likely to participate in activities such as watching television or listening to the radio and leisure walking [37, 38]. This may explain why the participants answered this question differently. Since age is one of the most commonly reported elements in clinical research studies, the distributions of this may need to be considered when interpreting the patient-reported outcomes using the PRWE in clinical studies of persons with wrist conditions. This also reflective of the nature of the DRF population, where fractures in younger persons are most likely high velocity related to sports and MVA, in comparison to older adults where low-velocity injuries such as falls from standing height predominate [41].

The strengths of the current study are its high PSI values and the excellent power of fit with a sample size of 382 patients. Although we used available data from a DRF population for this secondary analysis, we were able to examine for DIF based on gender, age, work status and hand dominance and affected side. In Rasch tradition, item and person measures are not considered sample-dependent if the data can be shown to fit the Rasch model after adjustment for DIF. We were able to build on previous work using RA to examine the predominantly similar outcome measure the PRWHE [16] while addressing the limitations of that previous study. While we anticipated measurement properties would be similar given the small variations between the scales, we now have empirical data confirming this. More importantly, our study addresses previously unreported measurement properties including the impact of the working status of the participants on the performance of the PRWE. Return to work is a considered a vital indicator of recovery [18, 19], this study utilized the current and pre-injury work status as a person factor for analyzing the measurement properties of PRWE. The ANOVA supports that even though the working and the non-working populations answered the question differently, still the mean total PRWE score of people who have returned to work (− 1.47 logits) and those not working (− 1.48 logits) have no statistical difference (F (1,379) = 0.033, *p* = 0.86).

The limitations of the current study are that the data were collected only from one location and at a single recovery time point. As this data represents participant status at 6 months post-injury, we might expect a floor bias, as persons are generally recovered and may have very low scores on the PRWE. However, this analysis suggests even at this lower end of scores, the data still fit the Rasch model. While RA supposes that results should be transferable across patient populations [9], our study does not address if similar findings would occur across diverse patient populations, or across a DRF population at different time points in recovery. Finally, the rescoring, splitting and bundling of items required to achieve model fit is likely not operational for general clinical practice, and future work should confirm our findings before undertaking the development of systems to facilitate Rasch scoring, such as specific conversion tables (Table 3) or digital apps.

**Table 3:** Transformation Matrix for Converting Raw Ordinal Level Scores to Interval-level Scores, Using the Revised Scoring Where the Scale is Out of a Maximum of 10 Points. This conversion can be used only with the modified PRWE questionnaire

ROW SCORE (ORDINAL)	LOGIT LOCATION	INTERVAL-LEVEL SCORE
0	−5.49	0.06
1	−4.49	4.34
2	−3.79	7.38
3	−3.30	9.50
4	−2.91	11.18
5	−2.58	12.60
6	−2.28	13.86
7	−2.01	15.03
8	−1.76	16.11
9	−1.53	17.13
10	−1.30	18.09
11	−1.09	18.99
12	−0.90	19.84
13	−0.71	20.64
14	−0.54	21.38
15	−0.37	22.09
16	−0.22	22.75
17	−0.08	23.37
18	0.05	23.95
19	0.18	24.50
20	0.30	25.02
21	0.41	25.52
22	0.52	26.00
23	0.63	26.46
24	0.73	26.91
25	0.84	27.35
26	0.94	27.79
27	1.04	28.23
28	1.15	28.68
29	1.25	29.14
30	1.36	29.62
31	1.48	30.13
32	1.60	30.65
33	1.73	31.22
34	1.87	31.82
35	2.03	32.48
36	2.19	33.20
37	2.38	34.02
38	2.61	34.97
39	2.88	36.15
40	3.24	37.69

## Conclusion

In conclusion, this Rasch analysis suggests the psychometric measurement and interval level properties of the PRWE are robust, reinforcing previous studies on PRWE/PRWHE’s psychometric properties using both classical test theory and Rasch approaches. The PRWE conformed to many of the fit expectations of the Rasch model, but not all. With modifications, we were able to fit all the items to the Rasch model.

## Additional file

Additional file 1: Patient Rated Wrist Evaluation. (DOCX 18 kb)

## Abbreviations

ANOVA: Analysis of Variance
CTT: Classical Test Theory
DASH: Disabilities of the Arm, Shoulder, and Hand
DIF: Differential Item Functioning
DRF: Distal Radius Fracture
IRT: Item Response Theory
LR: Likelihood Ratio
MVA: Motor Vehicle Accidents
PCA: Principal Components Analysis
PREE: Patient-Rated Elbow Evaluation
PROM: Patient-Reported Outcome Measure
PRWE: The Patient-Rated Wrist Evaluation
PRWHE: Patient-Rated Wrist and Hand Evaluation
PSI: Person Separation Index
RA: Rasch Analysis
