# Supplementary material for: Rasch analysis of the patient-rated wrist evaluation questionnaire
Source: Arch Physiother. 2018 Feb 26;8:5. doi: 10.1186/s40945-018-0046-z (PMC5828063; doi:10.1186/s40945-018-0046-z)
Supplement: Supplementary file 1 — Patient Rated Wrist Evaluation. (DOCX 18 kb) [file 40945_2018_46_MOESM1_ESM.docx]

**Appendix 1**

Name: Date:

**PATIENT RATED WRIST EVALUATION**

*The questions below will help us understand how much difficulty you have had with your wrist in the past week. You will be describing your* ***average*** *wrist symptoms* ***over the past week*** *on a scale of 0-10.* Please provide an answer for **ALL** questions. If you did not perform an activity, please **ESTIMATE** the pain or difficulty you would expect**.** If you have **never** performed the activity, you may leave it blank.

| 1. PAIN | |
| --- | --- |
| Rate the **average** amount of pain in your wrist over the past week by circling the number that best describes your pain on a scale from 0-10. A zero **(0)** means that you **did not** have any pain and a **ten (10**) means that you had the **worst pain you have ever experienced** or that **you could not do the activity because of pain.** | |
| RATE YOUR PAIN: Sample Scale *L* 0 1 2 3 4 5 6 7 8 9 10  No Pain Worst Ever | |
| At rest | 0 1 2 3 4 5 6 7 8 9 10 |
| When doing a task with a repeated wrist movement | 0 1 2 3 4 5 6 7 8 9 10 |
| When lifting a heavy object | 0 1 2 3 4 5 6 7 8 9 10 |
| When it is at its worst | 0 1 2 3 4 5 6 7 8 9 10 |
| How often do you have pain? 0 1 2 3 4 5 6 7 8 9 10  Never Always | |

| 2. FUNCTION | |
| --- | --- |
| A. SPECIFIC ACTIVITIES  *Rate the* ***amount of difficulty*** *you experienced performing each of the items listed below - over the past week, by circling the number that describes your difficulty on a scale of 0-10. A* ***zero*** *(0) means you did not experience any difficulty and a* ***ten*** *(10) means it was so difficult you were unable to do it at all*.  Sample scale *û* 0 1 2 3 4 5 6 7 8 9 10  No Difficulty Unable  To Do | |
| Turn a door knob using my affected hand | 0 1 2 3 4 5 6 7 8 9 10 |
| Cut meat using a knife in my affected hand | 0 1 2 3 4 5 6 7 8 9 10 |
| Fasten buttons on my shirt | 0 1 2 3 4 5 6 7 8 9 10 |
| Use my affected hand to push up from a chair | 0 1 2 3 4 5 6 7 8 9 10 |
| Carry a 10lb object in my affected hand | 0 1 2 3 4 5 6 7 8 9 10 |
| Use bathroom tissue with my affected hand | 0 1 2 3 4 5 6 7 8 9 10 |
| B. USUAL ACTIVITIES  *Rate the* ***amount of difficulty*** *you experienced performing your* ***usual*** *activities in each of the areas listed below, over the past week, by circling the number that best describes your difficulty on a scale of 0-10. By “usual activities”, we mean the activities you performed* ***before*** *you started having a problem with your wrist. A* ***zero*** *(0) means that you did not experience any difficulty and a* ***ten*** *(10) means it was so difficult you were unable to do any of your usual activities.* | |
| Personal care activities (dressing, washing) | 0 1 2 3 4 5 6 7 8 9 10 |
| Household work (cleaning, maintenance) | 0 1 2 3 4 5 6 7 8 9 10 |
| Work (your job or usual everyday work) | 0 1 2 3 4 5 6 7 8 9 10 |
| Recreational activities | 0 1 2 3 4 5 6 7 8 9 10 |

© JC MacDermid
